# Magnetic Nanoparticles for Targeting and Imaging of Stem Cells in Myocardial Infarction

**DOI:** 10.1155/2016/4198790

**Published:** 2016-04-03

**Authors:** Michelle R. Santoso, Phillip C. Yang

**Affiliations:** Division of Cardiovascular Medicine, Stanford University, Stanford, CA 94305, USA

## Abstract

Stem cell therapy has broad applications in regenerative medicine and increasingly within cardiovascular disease. Stem cells have emerged as a leading therapeutic option for many diseases and have broad applications in regenerative medicine. Injuries to the heart are often permanent due to the limited proliferation and self-healing capability of cardiomyocytes; as such, stem cell therapy has become increasingly important in the treatment of cardiovascular diseases. Despite extensive efforts to optimize cardiac stem cell therapy, challenges remain in the delivery and monitoring of cells injected into the myocardium. Other fields have successively used nanoscience and nanotechnology for a multitude of biomedical applications, including drug delivery, targeted imaging, hyperthermia, and tissue repair. In particular, superparamagnetic iron oxide nanoparticles (SPIONs) have been widely employed for molecular and cellular imaging. In this mini-review, we focus on the application of superparamagnetic iron oxide nanoparticles in targeting and monitoring of stem cells for the treatment of myocardial infarctions.

## 1. Introduction

Heart disease is the leading cause of death in men and women worldwide and kills more than 600,000 Americans annually [1]. Massive efforts are underway to treat heart failure, yet significant challenges exist due to the heart's inability to regenerate itself. Of the types of heart failure, the most common is coronary artery disease (CAD) where plaque builds up in the arteries supplying oxygen to the heart, resulting in cardiac ischemia and manifesting as a heart attack. On a cellular level, cardiac ischemia initiates a cascade of irreversible damage leading to cell death, regional contractile dysfunction, and progressive fibrosis of the heart with resultant scar tissue. Because the cardiomyocytes (the main building blocks of the heart tissue) cannot proliferate and replace damaged cells, the remnants of severe injuries like myocardial infarction [2] remain decades after the cardiac insult. During this disease process, cardiac progenitor cells (CPCs) have been reported to migrate to the injured site, differentiate into cardiomyocytes, and eventually regenerate the myocardium [3–6]. However, the native population of CPCs is extremely limited and decreases significantly during the aging process [7], compromising the myocardial repair potential.

To compensate for the lack of CPCs in the injured site, therapeutic delivery of autologous patient-specific human induced pluripotent stem cell- (iPSC-) derived cardiomyocytes (iCMs) has been proposed, generating promising outcomes in preclinical studies [8–11]. Although intense efforts are made to enhance the survival of transplanted iCMs, including the implementation of growth factor-supplemented collagen matrices [12], poor engraftment and nonsustained contractility of these cells are recognized as the major limitations to clinical translation [13, 14]. Another important clinical issue is the lack of suitable techniques for the monitoring of the viability of injected stem cells in the myocardium. Additionally, a few hours after their injection, less than 10% of transplanted stem cells remain at the target site [15]. Another obstacle to clinical translation of iCMs is the lack of suitable techniques to track injected stem cells in the myocardium. To overcome these issues while establishing safe and reliable iCM therapy, novel approaches are required to deliver a high concentration of cells into the injured tissue and monitor their engraftment and viability.

Nanomedicine, the integration of engineered nanoparticles (NPs) with therapeutic products and medical procedures, is a rapidly growing field. Of the NPs under development and research in biomedical applications, superparamagnetic iron oxide nanoparticles (SPIONs) are among the most widely employed and are being studied for pathology characterization (e.g., magnetic resonance imaging (MRI) and ultrasensitive diagnostic assays) and treatment (e.g., drug delivery, cancer treatments, specific cell delivery, and tracking) [16]. Their biocompatibility, superparamagnetic properties, nanorange size control (3 to several hundred nm), and tailor-made surface coating for selective binding (amongst other properties) enable precision biologics for site-specific delivery and monitoring of small molecules, drugs, and cells [17]. Due to their unique magnetic properties, SPIONs are suitable agents for* in vivo* monitoring of the stem cells. In this mini-review, we will cover the recent efforts to employ SPIONs in stem cell applications and assess the potentials of and challenges in this field.

## 2. Imaging Modalities for Stem Cell Monitoring

To evaluate the distribution and efficacy of stem cells in cardiac therapy, it is essential to track the fate of injected cells with various imaging modalities. Cell labeling is currently accomplished via introduction of the probes into the cells and/or integration of the reporter genes into the genome. The cell labeling strategy employs a multitude of imaging modalities available today, including magnetic resonance imaging (MRI), single photon emission computed tomography (SPECT), fluorescence imaging, bioluminescence imaging (BLI), and positron emission tomography (PET), to detect the signal [18].

**Figure 1:**
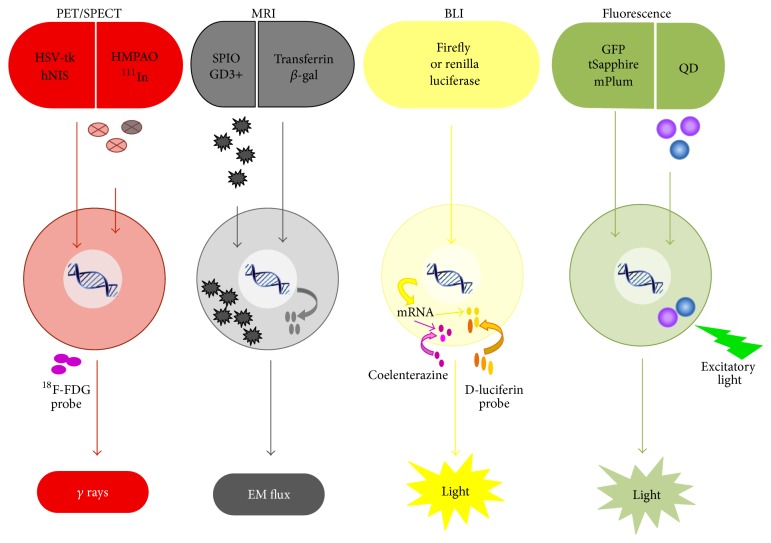
Schematic representation of cell labeling and imaging modalities (reproduced with permission [25]).

Such techniques have unique applications in stem cell therapy. Previous studies employed SPECT imaging to track radiolabeled cells (with 100 MBq 2-[^18^F]-fluoro-2-deoxy-d-glucose (^18^F-FDG)) in myocardial infarction models [19–21], while others utilized bioluminescence and fluorescence imaging to monitor the transplantation of the genetically modified stem cells to express luciferase and/or green fluorescent protein [22]. MRI allows high spatial resolution and signal persistence. This unique capability allowed long-term monitoring of cell migration in the heart and other tissues of the body [23, 24]. Although MRI has less sensitivity in comparison to other imaging modalities, the high temporal-spatial resolution and exquisite tissue contrast make MRI the most appropriate and widely advocated method for* in vivo* tracking of stem cells. The advantages/disadvantages and advances of these imaging modalities are discussed in the previous reviews [25, 26] and are schematically shown in Figure 1.

To date, however, no perfect imaging method exists with all the required features for clinical applications (e.g., high sensitivity and resolution, safety, targeting capability, and long durability). The integration of nanotechnology, and more specifically magnetic nanoparticles, is a rapidly emerging study in biomedicine to address the shortcomings of today's imaging technology.

## 3. Nanoparticles for Stem Cell Imaging

In order to visualize the stem cells under MRI, it is essential to label the cells with a suitable contrast agent. Contrast agents are indispensable materials to enhance the signal from specific cells* in vivo*.

**Figure 2:**
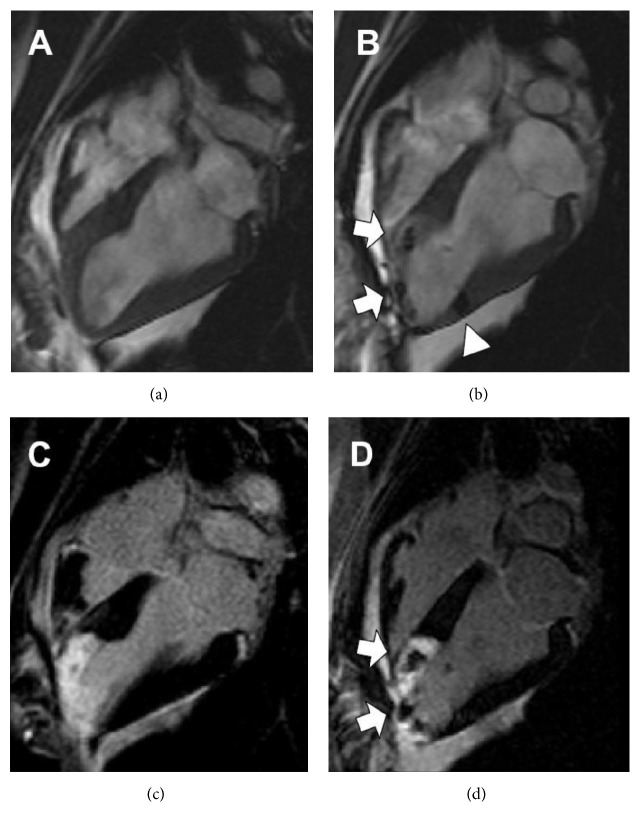
*In vivo* visualization immediately before and after IFP-MSC injection. Long-axis SSFP MRI view of left ventricle before (a) and after (b) transcatheter injection of 4 × 106 IFP-labeled MSCs into the infarct at the apex (arrows) and adjoining normal myocardium (arrowhead). Delayed enhancement MRI highlights areas of nonviable infarcted myocardium using the same view as above, before (c) and after (d) injection of IFP-labeled MSCs. MSCs appear dark against hyperenhanced infarct (reproduced with permission [27]).

Iron-containing particles have been used extensively for this purpose. For example, Hill et al. [27] labeled mesenchymal stem cells (MSCs) isolated from pigs with an iron fluorophore particle (IFP) for subsequent posttransplantation detection with MRI. They report that IFPs effectively and quickly labeled MSCs in a dose-dependent manner and the labeling procedure did not compromise the proliferation or differentiation capacity of the cells. MRI was performed at days 4, 8, and 21 after percutaneous injection of IFP-labeled MSCs to treat fresh myocardial infarcts in pigs. Histological examination detected the injection sites, which contained IFP-bearing MSCs and confirmed their long-term retention at the target. This method can track stem cells both* ex vivo* with fluorescence microscopy and* in vivo* with MRI shown in Figure 2.

Superparamagnetic iron oxide nanoparticles (SPIONs), a smaller variation of IFP, are one of the most employed contrast agents for the labeling of stem cells [16, 28]. Studies have pointed out their unique magnetic properties and excellent biocompatibility, demonstrating the potential for translational clinical studies. Hua et al. [29] used SPION-labeled bone marrow MSCs and successfully tracked the transplanted cells with MRI for different time courses. They demonstrated that the signal strength reduced over time whereas the signal region progressively expanded into the infarcted myocardium.

Long-term retention of transplanted cells is another important marker of the efficacy of stem cell therapy. The cells must be firmly engrafted in their target tissue and adapt to and remain viable in the complex host environment weeks and months after the injection. For imaging purposes, the cells must be fixed to contrast agents that are equally capable of staying in the recipient subject. This aspect is challenging as current popular contrast agents decay quickly; gadolinium, a contrast agent used prominently in MR imaging, has a half-life of approximately 1.5 hours. Due to their high persistence, SPIONs have gained traction as a long-lasting contrast agent in the context of cardiovascular applications and have thus emerged as a preferred labeling strategy for MR imaging. Multiple groups have tested their longevity. Blocki et al. utilized the SPION-labeled MSCs, which were encapsulated in collagen-based microcapsules and tracked after intramyocardial injection by MRI. The MSCs were progressively released from their microcapsules and the MR signals of encapsulated MSCs were monitored for several weeks [30]. In another study, adipose tissue-derived stem cells (ASCs) were encapsulated in magnetocapsules (semipermeable membrane microcapsules labeled with Endorem) and showed long-term viability and retention in myocardium [31]. Bai et al. [32] further demonstrated that lentivirus-mediated expression of GFP and luciferase allowed effective quantification with luciferase-based bioluminescence imaging and tracking with GFP, using human subcutaneous adipose tissue-derived cells (hASCs) injected into the mice myocardium. Findings from all aforementioned studies suggested that the injected stem cells remained viable for a long period of time (10 weeks) and did not distribute to other sites.

## 4. Challenges of Stem Cell Imaging with SPIONs

Despite existing successes with SPION-MRI monitoring, additional work is necessary to determine rigorously their efficacy in stem cell imaging both* in vitro* and* in vivo*. Yao et al. injected SPION-labeled endothelial progenitor cells (EPCs) into rat myocardium. MRI confirmed that iron-positive cells at the injection site 10 days later. The iron-positive signal persisted for two months. Upon closer examination, however, they found that the enhanced signal intensity detected was mainly attributed to macrophages that had consumed the SPION bound to dead cells [33]. Such reports were made throughout the literature. Monitoring of stem cell viability, which was thought to correlate with the strength of the SPION signal, was not feasible.

**Figure 3:**
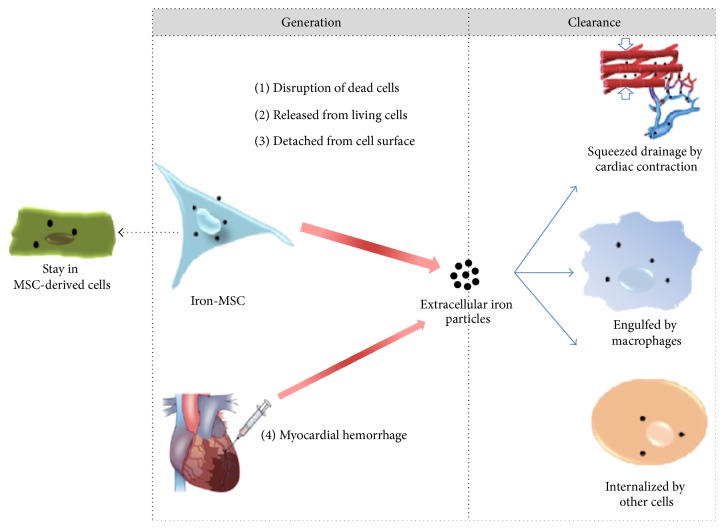
Schematic representation of generation and clearance of SPIONs in the myocardium (reproduced with permission [34]).

One of the main drawbacks associated with SPION is its inability to distinguish between viable and nonviable cells. Recently, Huang et al. [34] showed that the long lasting MR signals were mainly generated by extracellular, instead of intracellular, iron particles. The high concentrations of extracellular SPIONs arose from iron extruded from injected cells and compounded with the lack of significant iron clearance in the myocardium. In addition, long-term retention of iron nanoparticles in the myocardium may result in oxidative stress induction and consequently affect the cell viability. The mechanisms involved in the generation of extracellular SPIONs and clearance in the myocardium are shown schematically in Figure 3.

The application of MRI for longitudinal tracking of transplanted stem cells in a preclinical model of myocardial injury faces significant challenges due to existing controversies. Recently, Kim et al. [35] showed that the SPION-labeling of the stem cells cannot be useful for long-term follow-up of the injected cells in the myocardium. The study demonstrated the need to consider overestimation of cell survival and false positive signal. They suggested that SPION-labeling can be a suitable candidate to check the efficacy of initial cell injection into a target site and to concentrate the cells in the injection zone. Consistent with this study, Naumova et al. [36] demonstrated that SPION-labeling can be only used to probe the injection efficacy and particle localization.

**Figure 4:**
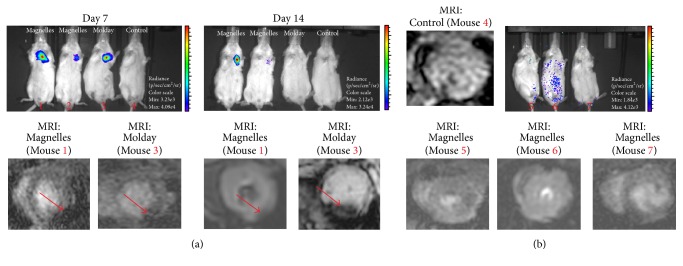
(a) Representative BLI images of the mice injected with Magnelle and Molday ION labeled reporter gene-transduced iCMs or PBS alone as control, after days 7 and 14 of cell injections (top) and their corresponding* in vivo* MRI images of the murine hearts (bottom); red arrows show the signal from the injected labeled cells. (b) Representative BLI images of the selected mice with dead cardiomyocytes (after day 14 of Magnelle labeled cells) and the corresponding* in vivo* MRI images.

To overcome this issue, our group employed a novel live-cell tracking approach by use of a living contrast agent derived from magnetotactic bacteria (Magnelle, Bell Biosystem, SF, CA) for labeling of stem cells. Our preliminary data show that cell labeling with this novel magnetotactic bacteria contrast agent is a robust and safe biological approach to track the viable cells. Specifically, we showed that the live contrast agent is cleared within 1 week of cell death; in contrast, our control SPIONs (Molday) remained more than 2 weeks after cell death (see Figure 4).

**Figure 5:**
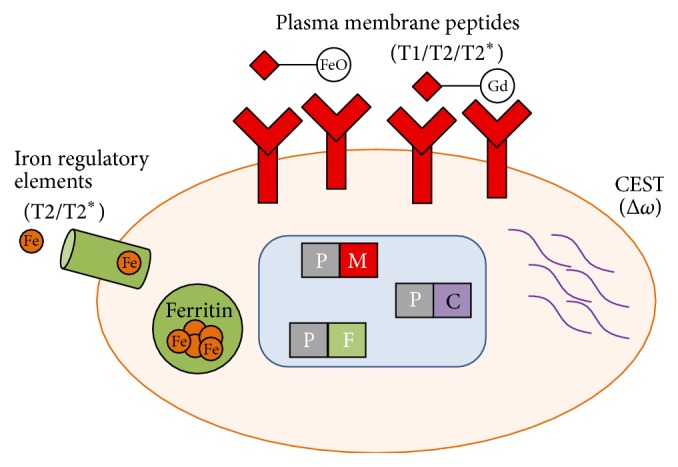
Schematic representation of MRI reporter genes used for cell labeling, reproduced with permission from reference [40].

Another major obstacle surrounding the SPION-labeling strategy is leakage of SPIO into adjacent cells (mainly through exocytosis process [37]) and advanced dilution after cell division and poor localization in the myocardial interstitial tissue [38, 39]. To overcome these challenges, the cells were labeled with MR reporter genes, which emerged as a new option to solve the problems associated with traditional cell tracking modalities [40]. The most common approach used for genetic labeling of the cells is the integration and subsequent expression of iron-controlling genes such as transferrin receptors and ferritin. Briefly, these cell membrane antigens or peptides specifically attach to SPIONs and the chemical exchange of saturated magnetization (CEST) reporter, producing CEST contrast in the cells (Figure 5).

Campan et al. [41] used human ferritin heavy chain (hFTH) as a reporter gene to follow stem cells in a rat myocardial infarction model. MRI tracking demonstrated that this system concurrently follows potential replication and differentiation of stem cells and alterations that occurred in the cardiac muscle. Another study showed that genetic manipulation of C2C12 cells expressing ferritin was capable of tracking the fate of live injected cells. The authors speculated that genetic labeling is a more beneficial approach for tracking transplanted stem cells compared to SPION-labeling [36]. In some studies, a combination of traditional imaging modalities and novel cell labeling/targeting approaches enabled more specific tracking of injected cells over a long period of time [42].

**Figure 6:**
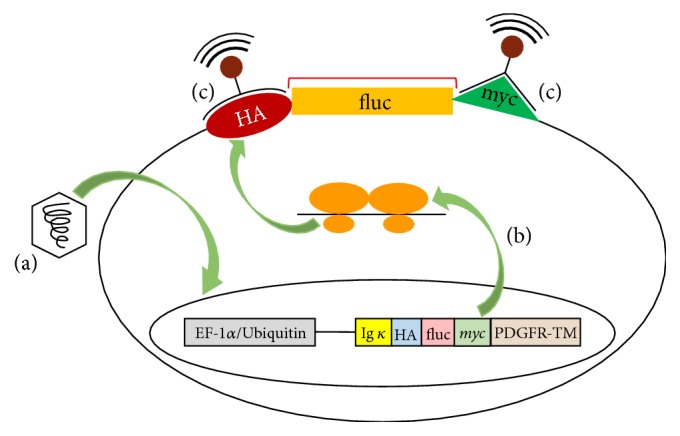
Mechanism of genetic manipulation of ESCs. HA, myc, and FLUC genes integrated into (a) the genome of ESCs and (b) coexpressed on the cell surface. SPIO conjugated monoclonal antibody specifically targets the antigens expressed on the cell surface, reproduced with permission from reference [42].

Chung and coworkers [42] used a new strategy to specifically detect live transplanted cells in the myocardium. They designed a new generation of reporter genes expressed as surface antigens, specifically hemagglutinin A, myc, and luciferase, on SPION-labeled embryonic stem cells (ESCs). SPIONs were conjugated to monoclonal antibodies that bound specifically to hemagglutinin A and myc antigens while luciferase was used to make a BLI signal. Through this strategy, the live transplanted ESCs were precisely tracked using MRI (Figure 6). Indeed, the combination of SPION-enhanced MRI and reporter genes with an immune-based approach is a useful option to overcome the challenges associated with current imaging modalities. Lee et al. [43] demonstrated that stem cells labeled with the adenovirus-mediated sodium iodide symporter (NIS) gene can be effectively and stably monitored in the infarcted myocardium of large animals (canines) using ^99m^Tc-TcO_4_^−^ SPECT imaging. Therefore, a blend of nuclear imaging with novel methods of cell labeling may provide the unprecedented improvement in stem cell tracking.

## 5. Nanoparticles for Stem Cell Delivery

To deliver the cells into the injured part of myocardium without open chest surgery, Cheng and coworkers [15] modified SPIONs, labeled cells, and injected them to random parts of the preinfarcted area. Specifically, they doubly conjugated an FDA-approved SPION (i.e., ferumoxytol, an intravenous iron product replacement used to treat anemia) with anti-CD45 (specific to exogenous bone marrow-derived stem cells) and with antibodies found in injured cardiomyocytes (myosin light chain). The dual antibody conjugated nanoparticles enabled high affinity binding of therapeutic cells to injured cardiomyocytes both* in vitro* and* in vivo*. The obtained results report that this approach can treat acute myocardial infarction.

**Figure 7:**
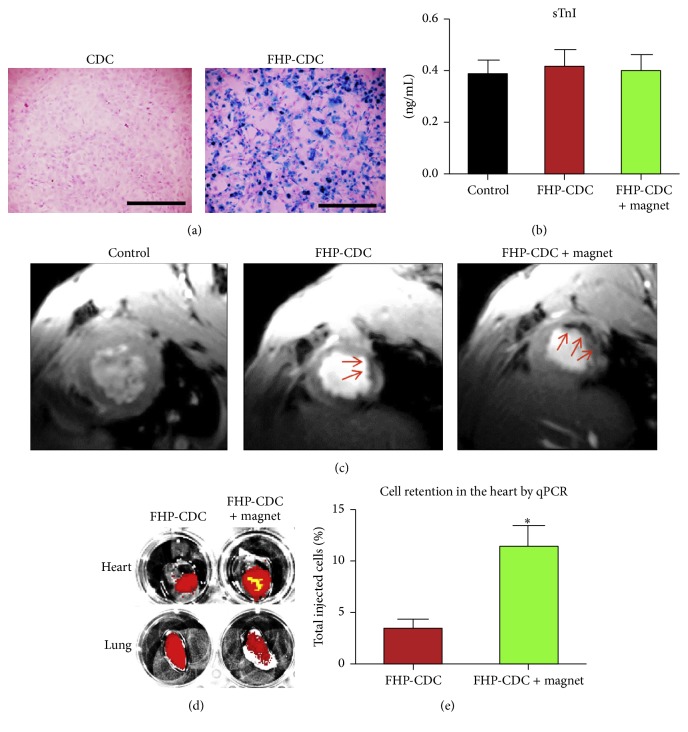
(a) Prussian blue staining showing the proper labeling of rat CDCs with ferumoxytol (FHB), (b) serum cardiac troponin-I (sTnI) values after intracoronary injection of 500,000 cells into noninfarcted rats, (c) T2^*∗*^ weighted MRI images showing higher cell retention in magnetic targeted samples (according to their dark signals which are shown by red arrows) 24 h after delivery of CDCs into rats myocardial infarcted model, (d) fluorescent imaging demonstrating the existence of labelled cells (in red, as the cells were transduced by red fluorescent protein) in rat in hearts and lungs, and (e) SRY qPCR quantitation of cell retention (reproduced with permission [44]). *∗* indicates statistical significance with *p* < 0.05.

In another study, Vandergriff and coworkers reported that heparin and protamine labeling significantly increased the percentage of ferumoxytol SPIONs taken up by cardiosphere-derived stem cells (CDCs) without cytotoxic consequence while changing the expression of only a handful of genes [44]. Thus considered a safe candidate for cardiac therapy, ferumoxytol-heparin-protamine labeled- (FHP-) CDCs were then injected via intracoronary route in ischemia/reperfusion (I/R) rats with and without a magnet placed over the rodent chest. The study demonstrated that the placement of* ex vivo* magnet significantly increased cell retention (Figure 7). Subsequent histological analyses concluded that FHP nanoparticles did not induce inflammatory or systemic iron overload responses in the myocardium. Additionally, rats treated with magnet-targeted FHP-CDCs had the most viable myocardium with the least scar tissue in the peri-infarct region. Echocardiographic analyses also demonstrated that this version of ferumoxytol design and delivery preserved the left ventricle ejection fraction (LVEF), providing the greatest evidence of cardioprotection, compared to both the control and the non-magnet-targeted FHP-CDC group. These studies underlie the potential for developing SPION technology for stem cell therapy of cardiac diseases.

Our group delivered human induced pluripotent stem cells- (iPSCs-) derived cardiomyocytes (iCMs) to the injured murine myocardium using dual conjugated SPIONs. We conjugated antibodies specific to injured cardiomyocytes (myosin light chain) and iCMs (VCAM) on the surface of SPIONs using the EDC (1-ethyl-3-(3-dimethylaminopropyl)carbodiimide) approach (unpublished data). Intravenous (IV) administration of the targeted SPIONS successfully delivered the iCMs to the injured myocardium. The theranostic efficacy of the SPIONs was analyzed* in vivo* by BLI to evaluate iCM engraftment and by MRI to localize the SPION-targeted iCMs and to evaluate the cardiac function. Multimodality assessment demonstrated successful delivery and engraftment of the iCMs up to day 28 and restoration of the myocardium.* In vivo* BLI signal decreased to background noise by day 14 in the control iCM arm while the iCMs in the targeted SPIONs arm demonstrated persistent engraftment signal up to day 28.

## 6. Challenges of Stem Cell Delivery with SPIONs

While SPIONs potentiate great advancement in the efficiency of stem cell delivery, additional studies must be done to further both cardiac stem cell research and SPION integration. For example, while Vandergriff et al. reported that ferumoxytol-labeling of cells improved cell uptake* in vivo* and were able to image the engrafted cells 3 days after injection [44], such a small time frame inadequately measures effectiveness of stem cell therapy. A study by Terrovitis et al. reported that a discontinued analog of ferumoxytol (ferumoxide) produced a positive signal 3 weeks after injection [39] but, as in the study by Yao et al.,* ex vivo* analyses determined that the signal was generated by macrophages after labeled cell death. Studies evaluating the use of ferumoxytol for cell-labeling should consider experiments to further characterize it, such as time-response experiments to determine if ferumoxytol is metabolized at the same rate as ferumoxide. Additionally, engraftment/viability and SPION signal strength are directly correlated with the number of cells that are injected into the myocardium. Though a larger number of transplanted cells may result in improved outcomes, myocardial injections consistently carry the risk of vascular embolism. Dose-response experiments for each SPION/cell pairing should be done to determine the opportunity cost of each injection volume.

Long-term viability of transplanted cells is a major hurdle in the advancement of stem cell therapy. The emergence of sensitive imaging techniques such as SPION-labeling has shown that transplanted stem cells are quickly targeted and obliterated by the host immune system. Extensive research is thus being done to find stem cells that will survive in the host myocardium, either by autologous, patient-specific induced pluripotent stem cells that can be differentiated into cardiac cells [45] or by isolating universally accepted sources of stem cells (e.g., from amniotic membrane derived mesenchymal stem cells [46]). Our group is currently researching safe gene-editing techniques to improve the longevity of patient-specific stem cell derived cardiomyocytes. Synergistic advancements in both imaging and isolation/gene editing of stem cells will in turn propel forward cardiac stem cell therapy forward.

## 7. Conclusion

Stem cells show promise for the treatment of the injured myocardium. To this end, researchers have sought new techniques to not only deliver stem cells into the desired region of the injured myocardium but also monitor their location, engraftment, proliferation, and viability. Among multiple imaging modalities, MRI allows a reliable and safe technique for stem cell tracking. Importantly, however, the sensitivity and success of MRI rely heavily on the contrast agent employed. Owing to their unique magnetic properties and excellent biocompatibility, superparamagnetic iron oxide nanoparticles (SPIONs) are recognized as one of the most promising and commercially available (e.g., ferumoxytol) contrast agents for stem cell labeling. There are exciting progresses in the field, with multiple labeling protocols, high efficiency production, and effective sensitivity for* in vivo* cell tracking to localize, target, and discriminate between live and dead cells. Despite these great advances in the field, many challenges still remain, including residual SPION signals after death of the labeled cells, variable longitudinal tracking duration, and the intracellular function of SPIONs to prevent dilution and loss of MRI contrast. Nonetheless, this quickly emerging field holds great clinical potential and further research should be pursued to rigorously outline the role of SPION-labeled stem cell therapy.
